# Comprehensive validation of the rapid tick exposure test (RaTexT®): accuracy, reproducibility and comparative performance to detect acaricide resistance in livestock ticks

**DOI:** 10.1186/s13071-026-07335-y

**Published:** 2026-03-24

**Authors:** Frans Jongejan, Laura Berger, Yakob Nagagi, Dennis Muhanguzi, Marisse van der Heide, Bjorn Wiggers, Luc Elders, Barbara Rauta de Avelar, Priscila Teixeira Ferreira, Guilherme Marcondes Klafke

**Affiliations:** 1 Department of Veterinary Tropical Diseases, Faculty of Veterinary Science, University of Pretoria, Private Bag X04, Onderstepoort, 0110 Republic of South Africa; 2 TBD International BV, Science Park 301, 1098 XH Amsterdam, The Netherlands; 3 Institute of Veterinary Clinical Sciences, National Taiwan University, Taipei, 106 Taiwan; 4 Tanzania Plant Health and Pesticides Authority (TPHPA), P.O. Box 3024, Arusha, Tanzania; 5 College of Veterinary Medicine Animal Resources and Biosecurity (COVAB), Makerere University, Kampala, Uganda; 6 Departamento de Parasitologia Animal, Instituto de Veterinária da Universidade Federal Rural do Rio de Janeiro, BR-465, Seropédica, Rio de Janeiro, Brazil; 7 Instituto de Pesquisas Veterinárias Desidério Finamor, Estrada do Conde, 6000, Eldorado do Sul, RS 92990-000 Brazil

**Keywords:** Rapid tick exposure test, Acaricide resistance, Ticks, Cattle, Pen-side test

## Abstract

**Background:**

RaTexT®, an innovative rapid tick exposure test, was recently developed to provide farmers with quick, on-site results to improve their acaricide resistance management. This was achieved by exposing partially engorged adult ticks to a specially designed acaricide-impregnated matrix fitted inside a transparent polypropylene box. Each RaTexT® box contains six strips of four small, interconnected compartments, in which ticks are exposed immediately after removal from cattle in the field. In this study, we assessed whether a single strip of four interconnected compartments, instead of six strips, was sufficient to accurately detect resistance to deltamethrin and to a combination of cypermethrin, chlorpyrifos and piperonyl butoxide (PBO). We also statistically analysed the optimal number of ticks per compartment (ranging between 5 and 8). Moreover, the test reproducibility was checked by two independent observers who counted dead and live ticks in each compartment. Finally, a comparative analysis was undertaken between adult ticks exposed in RaTexT® and in the adult immersion test (AIT), and also with larvae in the resistance intensity test (RIT) and in the larval packet test (LPT). The novelty of this study lies in comparing adult ticks exposed in RaTexT and in the AIT Test, thereby overcoming limitations of previous studies, in which adults in RaTexT were compared with larvae in the LPT.

**Methods:**

The internal coefficient of variation (CV) was calculated for each dose, box and acaricidal product to assess within-box consistency. The effect of the number of ticks per compartment (*n* = 5–8) was examined using Monte Carlo simulations. Inter-observer reliability of reading RaTexT® was statistically measured using Cohen’s kappa coefficient (*κ*). The comparative performance analysis of the bioassays was conducted using generalised linear models (GLMs) with laboratory and field strains of *Rhipicephalus microplus* ticks in Brazil.

**Results:**

Overall agreement between individual strips and their corresponding box classification was 91.3%, indicating high consistency between replicates. The predefined threshold of  ≥  90% accuracy was met by a single strip, supporting the test's robustness even with minimal replication. The internal coefficient of variation within each RaTexT® box was high for deltamethrin (1.285 at 1× dose, 1.109 at 5× dose and 1.268 at 10× dose), but lower for cypermethrin/chlorpyriphos/PBO (0.648 at 1, 0.305 at 5× dose and 0.194 at 10× dose). Variability in the controls was relatively high (CV 1.776). Monte Carlo simulations showed that diagnostic accuracy gradually increased from 81.2% with five ticks to 86.1% with eight ticks per compartment. Furthermore, there was substantial agreement between the mortality of ticks assessed by two independent observers (*κ* = 0.664). Finally, the comparative test analysis revealed that the deltamethrin resistance level in RaTexT® matched that observed in the AIT. Resistance to deltamethrin was also confirmed by the LPT, with resistance ratios (RR) of 33.8 and 39.5 for two different field strains (Biotech and UFRRJ, respectively). For cypermethrin/chlorpyriphos/PBO, RaTexT® exhibited significantly lower mortality than the AIT. Resistance was also confirmed by LPT, with RR of 5.2 for Biotech strain and 7.2 for the UFRRJ strain.

**Conclusions:**

Overall, these findings demonstrate that RaTexT® is accurate and reproducible with a single test strip, making it a practical and cost-effective test that complements traditional laboratory bioassays.

**Graphical Abstract:**

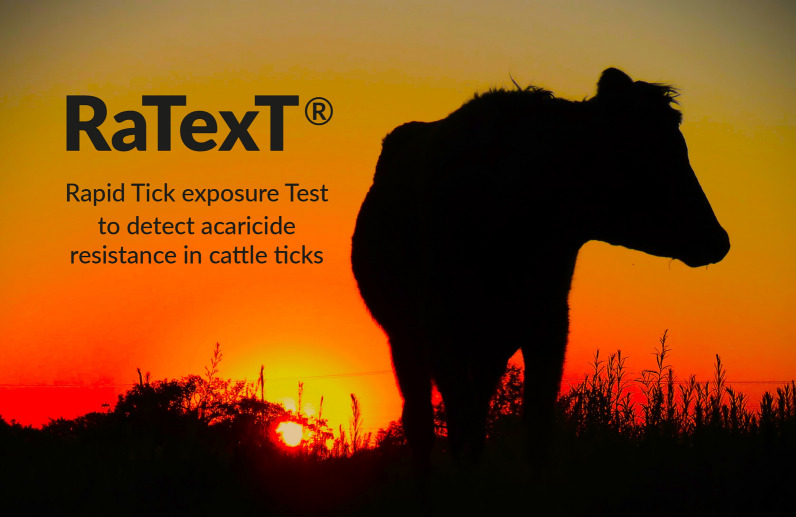

## Background

Ticks pose a significant obstacle to animal productivity through their feeding behaviour and ability to transmit a broad range of tick-borne pathogens to cattle, especially in tropical and subtropical regions [1]. Protozoan and rickettsial tick-borne diseases are prominent challenges for maintaining the health and management of cattle, affecting the livelihoods of farming communities in Africa, Latin America and Asia [2]. Controlling ticks and tick-borne diseases in cattle, particularly in the more susceptible *Bos taurus* breeds and crossbreeds, relies almost entirely on acaricides [3–5]. Although effective at killing ticks, many chemical acaricides are toxic, can leave residues in meat and milk and contribute to environmental pollution. The costs associated with their use impose a significant economic burden on the livestock industry. Most importantly, however, the frequent exposure of ticks to the same chemical classes has led to the development of acaricide resistance [6, 7] in sub-Saharan Africa ([8–10]), Latin America [11, 12], Asia [13, 14] and Australia [15].

Although acaricide resistance has been reported in a wide range of tick species, one-host *Rhipicephalus* ticks develop resistance more rapidly than other tick species because they are exposed to acaricide-treated cattle for at least 3 weeks. In Brazil, the one-host cattle tick, *Rhipicephalus microplus,* is widespread and often results in high tick burdens on cattle [12]. It first arrived in East and Southern Africa with the introduction of Asian cattle, where it replaced *Rhipicephalus decoloratus*, the native African blue tick [16]. It subsequently spread through most of Latin America and was introduced relatively recently into Ivory Coast with tick-infested cattle from Brazil, leading to widespread dissemination throughout West Africa [17]. In East Africa, *R. microplus* is well established in Tanzania [18], whereas in Uganda this tick has been discovered only relatively recently [19, 20]. Resistance of cattle ticks against synthetic pyrethroids, organophosphates and formamidines has been reported [8, 21]. Deltamethrin and cypermethrin are the main pyrethroids used for tick control on livestock, dominating the market in Tanzania (41%) and to a lesser extent in Uganda (20.4%). Chlorpyrifos is often combined with synthetic pyrethroids, representing approximately one third of the acaricidal market in both countries.

The Food and Agriculture Organisation (FAO) has recently issued guidelines on sustainable tick control and acaricide resistance management in livestock [22, 23]. Testing for acaricide resistance in cattle ticks is essential for applying resistance management strategies. The FAO has endorsed the larval packet test (LPT) since its publication in the* Plant Protection Bulletin* in 1971 [24], a decade after Stone and Haydock first described it in 1962 [25]. Other tests include the adult immersion test (AIT), larval immersion test (LIT) and the larval tarsal test (LTT) [26–31]. Both the LPT and AIT are the most common tests used for resistance diagnosis in laboratories worldwide [22]. However, the current gold-standard bioassays, particularly LPT and AIT, are not well suited for field diagnostics because they require specialised parasitological laboratory facilities and can take several weeks to complete. It has been recommended that rapid pen-side diagnostic tests be developed to differentiate resistance from malpractice in chemical tick control [22, 32].

Recently, we developed a novel rapid tick exposure test, termed the RaTexT®; TBD International BV, Utrecht, the Netherlands), where partly engorged adult ticks are exposed to a specially designed acaricide-impregnated matrix that provides rapid pen-side test results. This innovative concept was first tested in Brazil using synthetic pyrethroids, successfully differentiating between deltamethrin-resistant and -susceptible *R. microplus* ticks from reference laboratory colonies and field strains [33]. It also distinguished permethrin-resistant ticks from permethrin-susceptible ones in laboratory strains of *Rhipicephalus sanguineus* sensu lato [34]. In addition to its ability to quickly detect resistance as a point-of-care test, it has been used to introduce and adopt the resistance intensity protocol based on the latest WHO guidelines for resistance management of malaria mosquito vectors. This protocol utilises 1×, 5× and 10× higher concentrations to identify low, moderate and high levels of resistance, respectively, thereby increasing the practical value of RaTexT® as a decision-support tool. For example, a high level of synthetic pyrethroid resistance could prompt a change in acaricidal class, whereas a low level of resistance might not. Next, we modified the FAO-recommended LPT and introduced the resistance intensity test (RIT) [35], adopting the same resistance intensity protocol [36]. This allows direct comparison of resistance detection in adult ticks using RaTexT® with that in tick larvae via the RIT, applying the same acaricidal concentrations in both assessments. This approach has been successfully used to validate the diagnostic performance of RaTexT® for detecting acaricide resistance in cattle ticks in East Africa by comparing resistance levels identified in adult ticks using RaTexT® with those determined in larvae through the RIT [37].

In the present study, we evaluated the accuracy, reproducibility and comparative test performance of RaTexT® using a susceptible laboratory strain and two field strains of *Rhipicephalus microplus* ticks from Brazil. One of the aims was to determine whether a single strip with four interconnected compartments, instead of six strips, as in previous studies [34, 35], was sufficient to detect acaricide resistance accurately. The optimal number of ticks per compartment was also statistically assessed, and reproducibility was tested by two independent observers who evaluated tick mortality in each compartment of a RaTexT® box. Additionally, a comparative analysis was carried out between adult ticks exposed in the RaTexT® and AIT, as well as with larvae exposed in the RIT and LPT. A total of 63 RaTexT® boxes, each with six strips of four interconnected compartments impregnated with deltamethrin (*n* = 40) as well as cypermethrin/chlorpyriphos/piperonyl butoxide (PBO) (*n* = 23) were included in the study.

## Methods

### Ticks

A laboratory-reared colony of *R. microplus* known as the Porto Alegre (POA) strain was used as the reference population. This strain has been maintained at the Faculdade de Veterinária, Universidade Federal do Rio Grande do Sul (UFRGS), Porto Alegre (Brazil), in confined cattle without exposure to acaricides since its isolation in 1994. Field isolates of *R. microplus* were collected from cattle farms located in Seropédica, RJ (strain UFRRJ) and Guaíba, RS (strain Biotech), Brazil. Infested cattle were restrained in squeeze chutes and examined for the presence of ticks. Approximately 300 ticks (engorged and semi-engorged females) were manually removed and transferred to plastic containers. Twenty-five animals were inspected and sampled on each farm. Following collection, ticks were transported to the laboratory and divided into subsamples for evaluation with RaTexT® (semi-engorged females) and the AIT (fully engorged females). From each isolate, 30 engorged females were placed in plastic Petri dishes and incubated at 28 ± 1 °C and 80–90% relative humidity (RH) for 14 days to allow oviposition. After this period, the egg mass was gently mixed, and samples (500 mg) were transferred into 5-ml serum vials that were sealed with cotton plugs. The vials were incubated under the same conditions (28 ± 1 °C, 80–90% RH) until larval hatching. Larvae aged 14–21 days post-hatching were subsequently used in larval bioassays, including the RIT and the LPT.

### RaTexT® preparation and testing

The following commercial acaricide products were used in the tests: (i) deltamethrin (Vectocid®; Ceva Animal Health, Libourne, France) and (ii) a combination formulation containing cypermethrin and chlorpyrifos (Vectoclor® Plus Pour-on; Ceva Animal Health). The RaTexT® matrix was impregnated with each formulation at the manufacturer’s recommended concentration (1×), as well as at five- (5×) and 10-fold (10×) higher concentrations. For deltamethrin, the tested concentrations were 0.25, 1.25 and 2.5 mg/ml. For the cypermethrin/ chlorpyrifos/PBO combination, the tested concentrations were 1.5, 7.5, and 15 mg/ml, calculated based on the combined concentration of both active ingredients.

Each RaTexT® box contains six rows of four interconnected compartments where adult ticks are exposed to an acaricide-impregnated nylon matrix (Fig. 5). The nylon matrix was cut into the required shape using a laser-driven electric cutting machine (ScanNCut; Zijlstra, Groningen, the Netherlands) and then impregnated in a separate zip bag with a mixture of acaricides in acetone and olive oil (2:1) for each dilution. The control matrix was first impregnated with an acetone/olive oil diluent. Each acaricide mixture was thoroughly mixed with the matrix pieces inside the zip bag to ensure a uniform coating, following which the matrix pieces were dried overnight in an aluminium tray within a fume hood. Each matrix piece was secured to the bottom of each compartment of the RaTexT® container with a small drop of RTV-1 Elastosil E4 silicone glue (Wacker Chemie AG, Munich, Germany), applied to the bottom of the compartment. The inside of the lid remained uncovered. The boxes were stored at room temperature in the dark until the start of the test.

During testing, semi-engorged *R. microplus* females measuring 5–8 mm in length were chosen as the preferred size [38]. The collected ticks were placed in a plastic tray, allowed to move freely and selected based on sex, vitality (actively walking) and size. Excess moisture and debris were removed, negating the need for washing, as ticks must be dry before being introduced into the RaTexT® compartments. Using fine-tipped tweezers, eight semi-engorged females were carefully inserted into each compartment, beginning with the control and progressing to the highest acaricide concentration (10×). After loading, all lids were securely closed, and the RaTexT® box was placed inside a plastic zip bag containing a piece of moist tissue. Each box was kept at room temperature, out of direct sunlight, for 24–48 h.

After the incubation period, ticks were removed from the RaTexT® compartments, starting with the control group. The number of live and dead ticks was recorded using a standardised data capture form. A tick was considered dead, or "knocked down", if it was unable to walk. This assessment was made easier by placing each tick within a small grey circle printed on the form: ticks able to walk out of the circle were recorded as alive, while those that remained immobile—even after stimulation by breath and gentle prodding with blunt forceps—were classified as dead.

### Adult immersion test

The AIT was performed with modifications to the original protocol described by Drummond et al. [38]. Acaricide solutions were prepared by diluting the commercial formulations of deltamethrin (Butox® P CE25, 25 g/l; Merck Sharp & Dohme Saúde Animal LTDA, São Paulo, Brazil) and a combination of cypermethrin, chlorpyrifos and PBO (Cyperclor Plus, cypermethrin 150 g/l; chlorpyrifos 250 g/l; PBO 150 g/l; Ceva Santé Animale, Paulínia, Brazil). Three concentrations were tested: (i) the recommended dilution (RD) on the label (1× RD; 0.025 mg/ml for deltamethrin and 0.5 mg/ml for the cypermethrin/chlorpyrifos/PBO mixture); (ii) 5× and 10× the recommended dilution (5× CD and 10× CD, respectively). The emulsifiable concentrates were mixed with distilled water in 15-ml conical tubes to prepare the immersion solutions.

Groups of 10 engorged female ticks, sorted by weight to ensure homogeneity, were immersed in 10 ml of the respective solution for 5 min. Control groups were immersed in distilled water. After immersion, ticks were drained using a metal sieve, gently dried with paper towels and placed in plastic Petri dishes (90 × 10 mm). The dishes were incubated in an environmental chamber at 28 ± 1 °C and 80–90% RH. After 7 days, ticks that laid eggs were considered to be alive, while those that did not oviposit were classified as dead. After 14 days of treatment, the eggs from each dish were weighed and transferred to 10-ml glass assay tubes sealed with cotton plugs and incubated under the same conditions for 4 weeks to allow larval hatching. After this period, the percentage of larval hatching was visually estimated using a stereomicroscope to compare the proportion of empty eggs to the total egg mass. The same operator conducted all evaluations to ensure consistency. The experiments were performed in triplicate, with three independent groups of 10 females exposed to freshly prepared solutions for each treatment and control group. The in vitro efficacy was calculated according to Drummond et al. [38] using the following equations.

- $${\text{estimated reproduction }}\left( {{\mathrm{ER}}} \right) = \left( {{\text{g egg}}/{\text{ g female}}} \right) \, \times {\text{ estimated }}\% {\text{ hatch }} \times {2}0,000$$
- $$\% {\text{ control }} = \, \left[ {\left( {\Sigma {\text{ER untreated }}{-}\Sigma {\text{ER treated}}} \right) \, /\Sigma {\text{ER untreated}}} \right] \, \times { 1}00$$

### Resistance intensity test

The RIT was conducted using the same batches of commercial deltamethrin and the organophosphate–synthetic pyrethroid (OP + SP) combination products used in the RaTexT®[35], at the recommended concentration (1× dose) and at doses of 5× and 10× the recommended concentration. Briefly, stock solutions were prepared in acetone and olive oil (2:1), and 0.9 ml of each dilution was evenly applied to Whatman no. 1 filter papers (10 × 7 cm; Merck Life Science, Darmstadt, Germany) in triplicate. After drying for at least 60 min in a fume hood, the papers were stored in sealed plastic bags at room temperature until use. Larvae from each isolate (POA, Biotech and UFRRJ) were transferred (approx. 100 per packet) onto the impregnated papers, which were folded, clipped and incubated at 28 ± 1 °C and 80–90% RH. Mortality was measured after 24 h, starting with the control packets and moving to higher concentrations. Larvae that could not walk, even after CO₂ stimulation, were considered to be dead. Three replicates per concentration, along with untreated controls, were tested for each isolate.

### Larval packet test

The LPT was conducted following standard procedures [22, 23]. In brief, serial dilutions of deltamethrin (0.01, 0.02, 0.03, 0.05, 0.08, 0.13, 0.22, 0.36 mg/ml for the susceptible strain and 0.8, 1.3, 2.2, 3.6, 6, 10 mg/ml for the resistant strains) and of a cypermethrin/chlorpyrifos/PBO combination (0.56, 1.13, 2.25, 4.5, 9, 18, and 36 mg/ml) were prepared using a solvent mixture of trichloroethylene and olive oil (2:1). The impregnation of filter papers, packet preparation, larval exposure, incubation and mortality assessment were carried out identically to the procedures described for the RIT (section Resistance intensity test). Each test was performed in triplicate at all concentrations.

### Statistical analysis

All statistical analyses were carried out using Python 3.11 (2023; Python Software Foundation, Wilmington, DE, USA), the StatsModels library in Python and Polo-Plus software (Leora Solutions, Kerala, India).

#### Agreement and accuracy

To evaluate the consistency between individual strip results and the final classification of RaTexT® boxes, we analysed data from multiple *R. microplus* and *R. decoloratus* populations previously tested in Brazil and East Africa [33, 37]. Each RaTexT® box included six independent strips (internal replicates), each producing a categorical resistance classification (susceptible, low, moderate or high). The box-level classification, which served as the reference standard, was based on the combined results of all six strips. Agreement between single-strip results and the box classification was first assessed using overall accuracy.

To evaluate the robustness of the RaTexT® diagnostic performance, we performed two complementary bootstrap analyses (each with 1000 replicates). Simulated classifications were initially generated by randomly selecting subsets of one to six strips per box, with the final category determined by majority rule. Accuracy was computed as the proportion of simulated classifications matching the reference, which enabled the identification of the minimum number of strips needed to attain ≥ 90% accuracy. Simultaneously, a Monte Carlo resampling analysis was used to assess the impact of the number of individuals per compartment, based on the observed mortality probabilities at each concentration (1× , 5× , 10×). For scenarios with five, six, seven or eight ticks per compartment, 1000 bootstrap datasets were created, and strip-level classifications were compared to the corresponding box classification [37]. For each number of strips or ticks, mean accuracy and standard deviation (SD) were calculated to measure the stability of diagnostic performance under reduced replication or sample size.

Within-box variability was evaluated by calculating the internal coefficient of variation (CV) for each tested dose (control, 1× RD, 5× CD, 10× CD) within every RaTexT® box. Only the six individual strips (A–F) were included in this calculation; the overall box classification was not considered. For each dose, CV was calculated as the standard deviation divided by the mean mortality percentage across the six strips. Boxes with < 2 valid mortality values for a specific dose were omitted from that dose-specific CV calculation. The average internal CV per box was then determined by averaging the CV values across all valid doses. These figures were subsequently summarised by acaricide and dose to facilitate comparison of within-box reproducibility between treatments.

#### Reproducibility

To evaluate the reproducibility of categorical resistance classifications, the Brazilian tick mortality data were independently assessed strip by strip within each RaTexT® box by two trained observers using standardised criteria. Classifications were categorised into four distinct groups: susceptible, low resistance, moderate resistance and high resistance. For each box, the overall percentage agreement (the proportion of identical classifications) was calculated, along with Cohen’s kappa coefficient (*κ*) to account for agreement expected by chance [39]. Additionally, Gwet’s AC1 statistic was computed as a more robust measure in cases of unbalanced category prevalence [40].

#### Comparative test performance

Mortality data were analysed using binomial generalised linear models (GLM) [41] [42] to compare the performance of the RaTexT®, RIT and AIT across doses and populations. Models were fitted separately for deltamethrin and cypermethrin/chlorpyrifos/PBO, with AIT, the 10× discriminating dose and the susceptible reference strain POA set as reference categories. Odds ratios (OR) with 95% confidence intervals (CI) were derived from model coefficients. This approach allowed for the simultaneous assessment of test type, dose and population effects on mortality outcomes, while enabling direct comparisons with the susceptible reference strain. Mortality bar plots for the three tests were generated using Microsoft Excel (Microsoft Corp., Redmond, WA, USA), with the mean mortalities and standard deviations obtained for each concentration, test, isolate and acaricide.

#### Determination of the lethal concentration causing 50% mortality with the LPT

For the LPT, mortality data were analysed using probit regression with Polo-Plus software (version 1.0; LeOra Solutions). The parameters estimated for each assay included the lethal concentration causing 50% mortality (LC_50_), the slope of the regression line and the corresponding 95% CIs. Resistance ratios (RR) were calculated as the ratio of the LC_50_ of the tested population to that of the susceptible Porto Alegre reference strain . Differences between populations were considered significant when the 95% CIs of the estimates did not overlap.

Part of the deltamethrin mortality data used for RaTexT® comparisons were previously published for Brazilian populations [34, 35] and are reused here to allow direct comparison with newly generated AIT and RIT datasets and to support additional analyses, including single-strip validation, Monte Carlo simulations and GLM-based comparative performance.

## Results

**Fig. 1:**
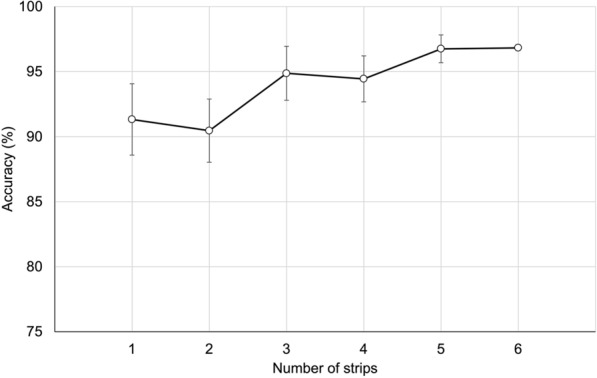
Bootstrap-estimated accuracy (1000 replicates) of the rapid tick exposure test (RaTexT®) resistance classification depending on the number of strips used per box. Accuracy was determined by the proportion of boxes where the simulated classification, based on the most frequent category among the selected strips, matched the reference classification derived from all six strips. Error bars indicate the standard deviation across bootstrap replicates

**Fig. 2:**
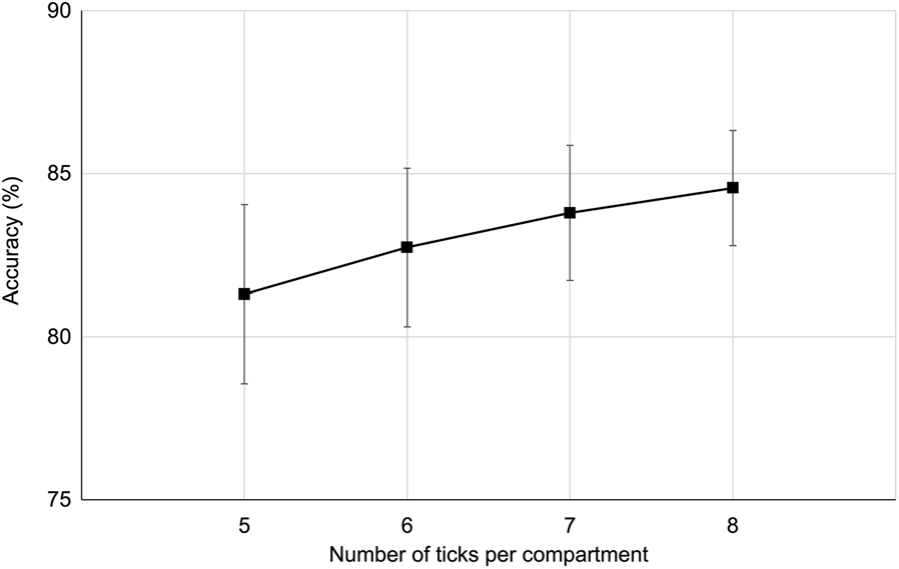
Impact of the number of ticks per compartment on the diagnostic accuracy of the rapid tick exposure test (RaTexT®). Results of 1000 bootstrap simulations illustrating the mean accuracy (points) and standard deviation (error bars) for different tick counts per compartment (*n* = 5–8)

Overall agreement between individual strips and the corresponding box classification was 91.3%, demonstrating high consistency between replicate results. Bootstrap simulations (1000 replicates) showed that using a single strip per box yielded a mean accuracy of 91.3% (SD 2.74%), while using two strips and three strips yielded a mean accuracy of 90.5% (SD 2.43%) and 94.9% (SD 2.07%), respectively. Furthermore, the mean accuracy was 94.4% (SD 1.77%) with four strips, 96.8% (SD 1.07%) with five strips and ultimately 96.8% (SD 0.00%) with all six strips (Fig. 1). Complementary Monte Carlo simulations, assessing the effect of the number of ticks per compartment (*n* = 5–8), indicated that the diagnostic accuracy of RaTexT*®* increased gradually from 81.2% (SD 2.4%) with five ticks per compartment to 86.1% (SD = 2.0%) with eight ticks per compartment (Fig. 2).

The internal CV calculated for each dose, box and acaricidal compound demonstrated consistency within each box. For deltamethrin, mean CVs were high at 1.285, 1.109 and 1.268 for the 1×, 5× and 10× doses, respectively. For cypermethrin/chlorpyriphos/PBO, the mean CV across boxes was 0.648 at the 1× dose, 0.305 at the 5× dose and 0.194 at the 10× dose. There was relatively high variability in the control group (CV 1.776). The test's reproducibility was further evaluated by two observers who counted dead and live ticks in each compartment using the walking criterion for mortality. The mean percentage agreement between the independent observers at the strip level was 83.3%, with a Cohen’s kappa coefficient averaging 0.664, indicating substantial agreement. Similarly, inter-observer reliability (Gwet’s AC1 statistical coefficient) was 0.707, indicating substantial agreement.

**Fig. 3:**
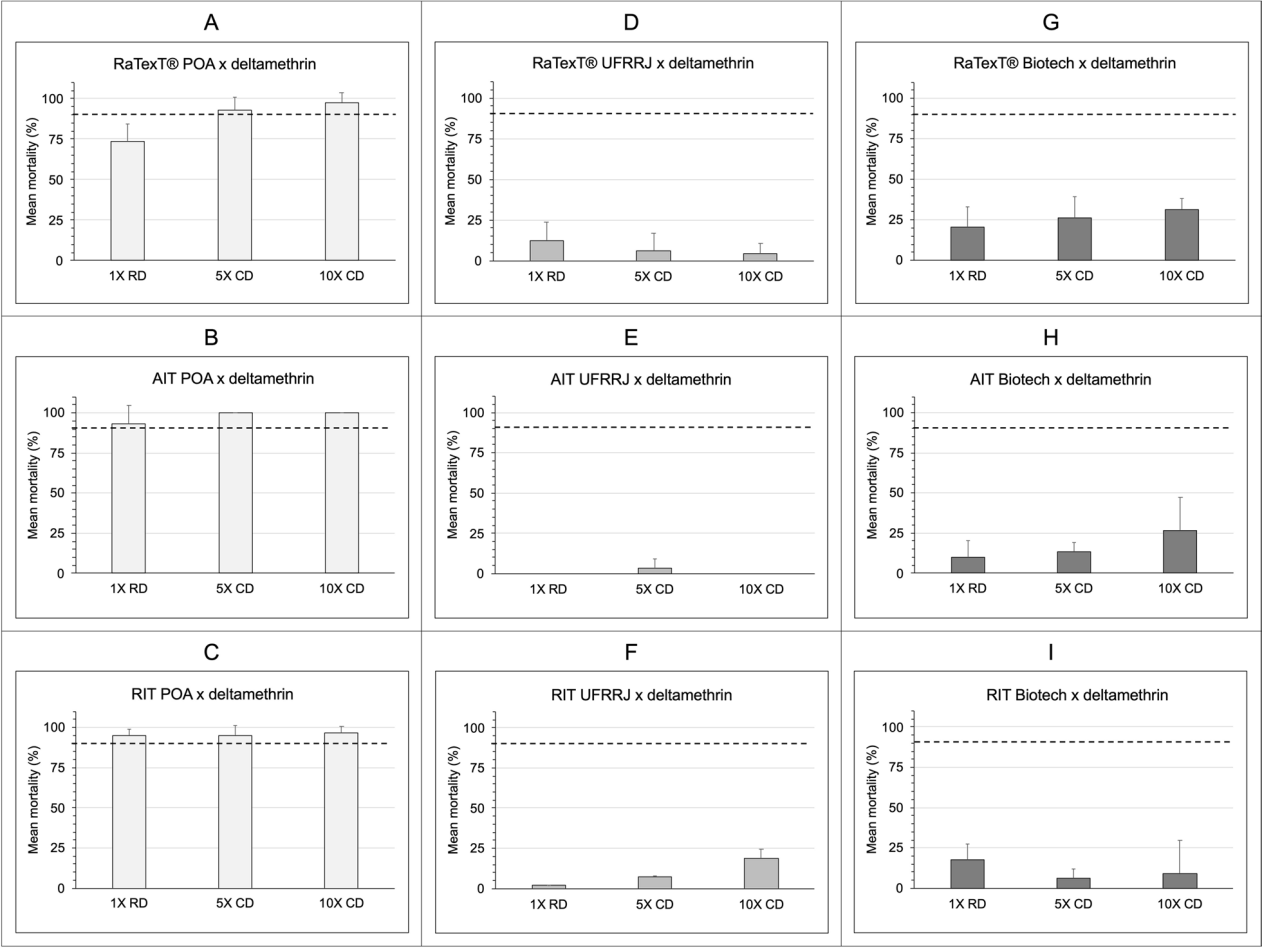
Comparative performance of the RaTexT®, AIT and RIT with deltamethrin formulation. Mortality of *Rhipicephalus microplus* ticks from Brazil assessed by the RaTexT® (top row), AIT (middle row) and RIT (bottom row) at discriminating concentrations. Columns represent the tick strains used: susceptible laboratory-reared strain POA and the field-collected resistant strains UFRRJ and Biotech. The dashed line indicates the 90% mortality threshold used for resistance classification. AIT, Adult immersion test; POA Porto Alegrea strain; RaTexT®, rapid tick exposure test; RIT, resistance intensity test; 1×, manufacturer’s recommended concentration; 5×, 10×, 5- and 10-fold higher concentrations

The comparative performance analysis of RaTexT®, AIT and RIT with the deltamethrin formulation revealed that the level of resistance to deltamethrin detected in the RaTexT® matched that observed in the AIT and RIT (Fig. 3). The GLM showed no significant differences in mortality between the RaTexT® and AIT (OR 1.42, 95% CI 0.80–2.52; *P* = 0.231), between the RIT and AIT (OR 1.14, 95% CI 0.70–1.86; *P* = 0.594) or between the RaTexT® and RIT (OR 1.26, 95% CI 0.88–1.83; *P* = 0.210) (Table 1). There were clear dose effects, with significantly lower odds of mortality at the 1× (OR 0.33, 95% CI 0.24–0.46; *P* < 0.001) and 5× (OR 0.48, 95% CI 0.36–0.65; *P* < 0.001) doses compared to the 10× dose. Both resistant field strains (Biotech and UFRRJ) exhibited markedly reduced odds of mortality relative to the susceptible reference POA strain (*P* < 0.001) (Table 1).

**Table 1:** Odds ratios and 95% confidence intervals) were estimated using a binomial generalised linear models

Acaricide	Comparison^a^	Odds ratio^b^	95% confidence interval	*P*-value
Deltamethrin	RIT vs AIT	1.14	0.70–1.86	0.594
	RaTexT vs AIT	1.42	0.80–2.52	0.231
	RaTexT vs RIT	1.26	0.88–1.83	0.210
	1× vs 10×	0.33	0.24–0.46	< 0.001
	5× vs 10×	0.48	0.36–0.65	< 0.001
	Biotech vs POA	0.0036	0.0023–0.0055	< 0.001
	UFRRJ vs POA	0.0023	0.0015–0.0035	< 0.001
Cypermethrin/ chlorpyriphos/PBO	RIT vs AIT	3.01	1.84–4.94	< 0.001
	RaTexT vs AIT	0.17	0.098–0.28	< 0.001
	RaTexT vs RIT	0.029	0.017–0.050	< 0.001
	1× vs 10×	0.06	0.036–0.10	< 0.001
	5× vs 10×	0.50	0.29–0.85	0.015
	Biotech vs POA	0.032	0.012–0.085	< 0.001
	UFRRJ vs POA	0.0093	0.0034–0.025	< 0.001

Analyses were carried out separately for deltamethrin and cypermethrin/chlorpyrifos/PBO

*AIT* Adult immersion test, *Biotech* *Rhipicephalus microplus* strain collected from Guaíba, RS (Brazil),* PBO* piperonyl butoxide,* POA *laboratory-reared *R. microplus* Porto Alegre strain (reference strain),* RIT* resistance intensity test,* UFRRJ* *R. microplus* strain collected from Seropédica, RJ (Brazil)

^a^1×, Manufacturer’s recommended concentration; 5× and 10×, 5- and 10-fold higher concentrations

^b^Odd ratio values < 1 indicate lower odds of mortality relative to the reference category; values > 1 indicate higher odds

**Fig. 4:**
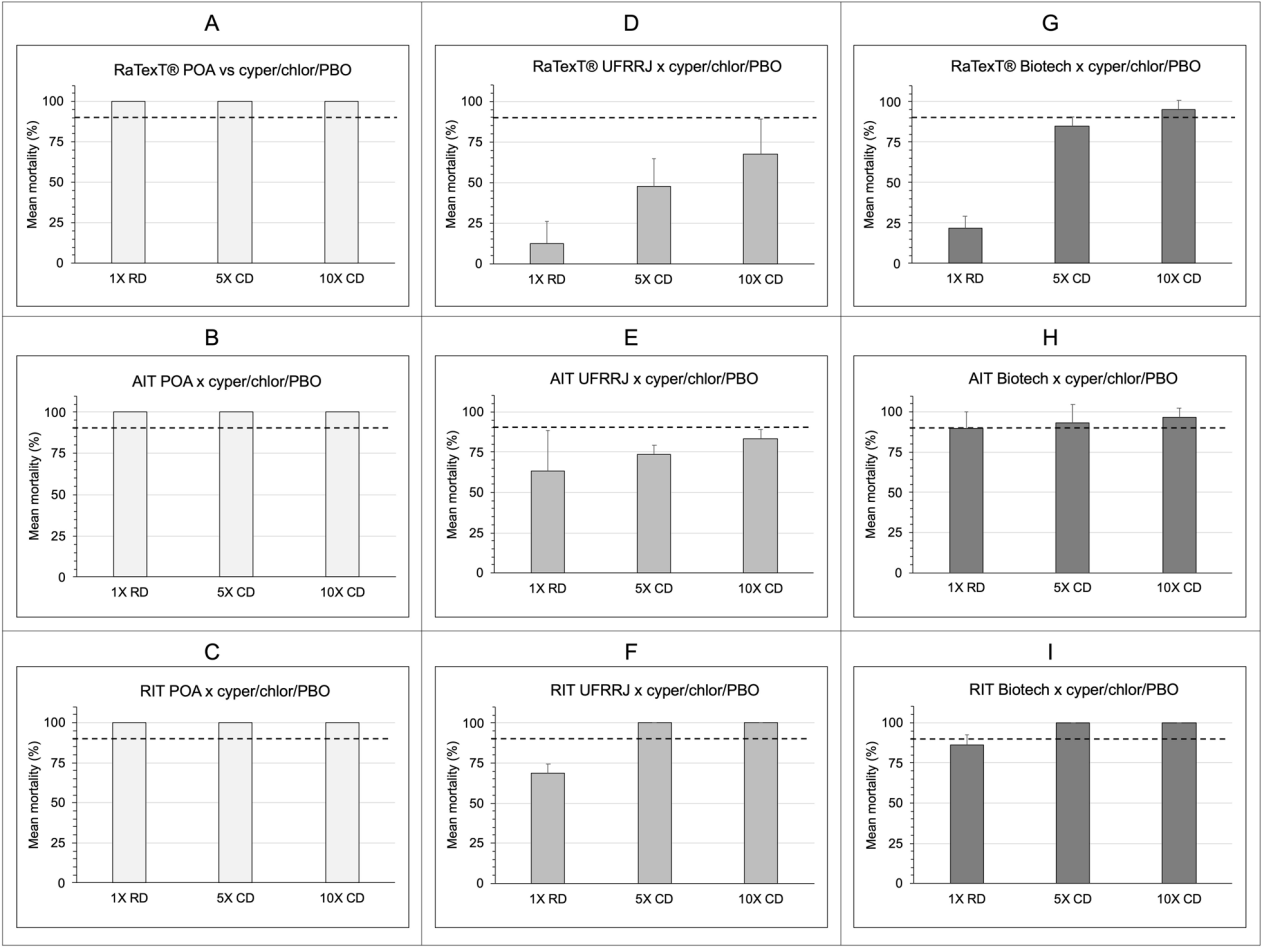
Comparative performance of the RaTexT®, AIT and RIT with cypermethrin/ chlorpyriphos/BPO. Mortality of *Rhipicephalus microplus* ticks from Brazil assessed by the RaTexT® (top row), AIT (middle row) and RIT (bottom row) at discriminating concentrations. Columns represent the tick strains used: susceptible laboratory-reared strain POA and the field-collected resistant strains UFRRJ and Biotech. The dashed line indicates the 90% mortality threshold used for resistance classification. AIT, Adult immersion test; BPO, piperonyl butoxide; POA Porto Alegrea strain; RaTexT®, rapid tick exposure test; RIT, resistance intensity test; 1×, manufacturer’s recommended concentration; 5×, 10×, 5- and 10-fold higher concentrations

The comparative performance analysis of the RaTexT®, AIT, and RIT with cypermethrin/chlorpyriphos/PBO revealed that the resistance level measured in the RaTexT® did not match those observed in the AIT and RIT (Fig. 4). The RaTexT® exhibited significantly lower mortality compared to the AIT (OR 0.17, 95% CI 0.098–0.28; *P* < 0.001) and the RIT (OR 0.029, 95% CI 0.017–0.05; *P* < 0.001), and the RIT showed a considerably higher tick mortality compared to the AIT (OR 3.01, 95% CI 1.84–4.94; *P* < 0.001). Similar to deltamethrin, dose effects were notable, with odds of mortality at the 1× (OR 0.06, 95% CI 0.036–0.10; *P* < 0.001) and 5× (OR 0.50, 95% CI 0.29–0.85; *P* = 0.015) doses being significantly lower than that at the 10× dose. Both field populations (Biotech and UFRRJ) demonstrated substantially lower mortality than the POA (*P* < 0.001) (Table 1).

Resistance to deltamethrin was confirmed through the LPT, which revealed RR of 33.8 and 39.5 for the *R. microplus* Biotech and UFRRJ field strains, respectively, when compared to the susceptible *R. microplus* POA reference strain. Resistance to cypermethrin/chlorpyriphos/PBO was also identified by the LPT, showing moderate resistance levels relative to the susceptible POA strain: RR of 5.2 and 7.2 for the Biotech and UFRRJ strains, respectively (Table 2).

**Table 2:** Larval packet test results showing lethal concentration values, slopes, 95% confidence intervals, and resistance ratios of *Rhipicephalus microplus* ticks from Brazil determined for deltamethrin and cypermethrin/chlorpyrifos/piperonyl butoxide

*Rhipicephalus microplus* strain^a^	Acaricide	*N* larvae	Slope	LC_50_ (95% CI)	Resistance ratio
POA	Deltamethrin	3166	3.386 ± 0.122	0.075 (0.065–0.086)	NA
	Cypermethrin/chlorpyriphos/PBO	2250	6.084 ± 0.327	0.796 (0.764–0.828)	NA
Biotech	Deltamethrin	1270	4.152 ± 0.192	2.537 (2.327–2.763)	33.8
	Cypermethrin/ chlorpyriphos/PBO	1919	3.330 ± 0.197	4.176 (3.577–4.770)	5.2
UFRRJ	Deltamethrin	1615	2.537 ± 0.128	2.959 (2.677–3.256)	39.5
	Cypermethrin/ chlorpyriphos/PBO	2157	3.883 ± 0.204	5.760 (5.339–6.198)	7.2

*LC*_*50*_ Lethal concentration causing 50% mortality,* NA* data not available,* PBO* piperonyl butoxide,* RR* resistance ratio

^a^See footnote to Table 1 for description of *Rhipicephalus microplus* strains

In the AIT, near-complete inhibition of oviposition and egg hatching was observed in the susceptible *R. microplus* POA strain at the 1× dose, confirming the full efficacy of both acaricidal compounds under controlled laboratory conditions. In contrast, the field-collected *R. microplus* strains Biotech and UFRRJ exhibited high reproductive output and hatchability even at the highest deltamethrin dose, indicating strong resistance (Table 3). For cypermethrin/chlorpyrifos/PBO, both the Biotech and UFRRJ strains displayed values indicating susceptibility to these compounds (Table 4).

**Table 3:** Adult immersion test results for *Rhipicephalus microplus* ticks exposed to deltamethrin at the manufacturer’s recommended concentration and 5- and 10-fold higher concentrations

Strain^a^	Group^b^	Female weight (mg)	Egg weight (mg)	Hatch (%)	Estimated reproduction (number of eggs)	% Control
POA	Control	3.05 ± 0.55	1.29 ± 0.34	90 ± 5	754,208	NA
	Deltamethrin 1×	3.05 ± 0.55	0.08 ± 0.14	6.7 ± 11.5	10,458	98.61%
	Deltamethrin 5×	3.05 ± 0.55	0	NA	0	100%
	Deltamethrin 10×	3.05 ± 0.55	0	NA	0	100%
Biotech	Control	2.05 ± 0.01	1.09 ± 0.12	95 ± 0	1,007,154	NA
	Deltamethrin 1×	2.05 ± 0.01	0.91 ± 0.12	93.3 ± 2.9	830,407	17.55%
	Deltamethrin 5×	2.05 ± 0.01	0.88 ± 0.01	91.7 ± 2.9	784,228	22.13%
	Deltamethrin 10×	2.05 ± 0.01	0.69 ± 0.12	90 ± 0	605,854	39.85%
UFRRJ	Control	2.28 ± 0.10	1.174 ± 0.079	91.66 ± 2.36	943,297	NA
	Deltamethrin 1×	2.55 ± 0.17	1.265 ± 0.106	72.33 ± 14.06	725,712	23.07%
	Deltamethrin 5×	2.44 ± 0.33	1.130 ± 0.186	86.67 ± 11.79	815,875	13.51%
	Deltamethrin 10×	2.46 ± 0.16	1.284 ± 0.032	83.33 ± 6.24	874,801	7.26%

Values are presented at mean (± standard deviation where appropriate) values of female and egg weights, hatchability, estimated reproduction and percentage control for each strain

* NA* Data not available

^a^See footnote to Table 1 for description of *Rhipicephalus microplus* strains

^b^1×, Manufacturer’s recommended concentration; 5× and 10×, 5- and 10-fold higher concentrations

**Table 4:** Results of the adult immersion test for *Rhipicephalus microplus* treated with cypermethrin/chlorpyrifos/piperonyl butoxide at the manufacturer’s recommended concentration and 5- and 10-fold higher concentrations

Strain^a^	Group^b^	Female weight (mg)	Eggs weight (mg)	Hatch (%)	Estimated reproduction (number of eggs)	% Control
POA	Control	3.05 ± 0.55	1.29 ± 0.34	90 ± 5	754,208	NA
	Cyper/chlor 1×	3.05 ± 0.55	0	NA	0	100%
	Cyper/chlor 5×	3.05 ± 0.55	0	NA	0	100%
	Cyper/chlor 10×	3.05 ± 0.55	0	NA	0	100%
Biotech	Control	2.05 ± 0.01	1.09 ± 0.12	95 ± 0	1,007,154	NA
	Cyper/chlor 1×	2.05 ± 0.01	0.09 ± 0.08	33.3 ± 38.2	37,398	96.29%
	Cyper/chlor 5×	2.05 ± 0.01	0.09 ± 0.16	16.7 ± 28.9	43,902	95.64%
	Cyper/chlor 10×	2.05 ± 0.01	0.04 ± 0.06	16.7 ± 28.9	17,886	98.22%
UFRRJ	Control	2.283 ± 0.085	1.174 ± 0.079	91.67 ± 2.36	943,297	NA
	Cyper/chlor 1×	2.257 ± 0.247	0.108 ± 0.038	38.33 ± 27.18	44,222	95.31%
	Cyper/chlor 5×	2.216 ± 0.216	0.306 ± 0.162	35.00 ± 21.60	121,858	87.08%
	Cyper/chlor 10×	2.508 ± 0.037	0.287 ± 0.029	33.33 ± 13.12	78,110	91.27%

Values are presented at mean (± standard deviation where appropriate) values of female and egg weights, hatchability, estimated reproduction and percentage control for each strain

*Chlor* Chlorpyrifos,* Cyper* cypermethrin,* NA* data not available

^a^See footnote to Table 1 for description of *Rhipicephalus microplus* strains

^b^1×, Manufacturer’s recommended concentration; 5× and 10×, 5- and 10-fold higher concentrations

## Discussion

**Fig. 5:**
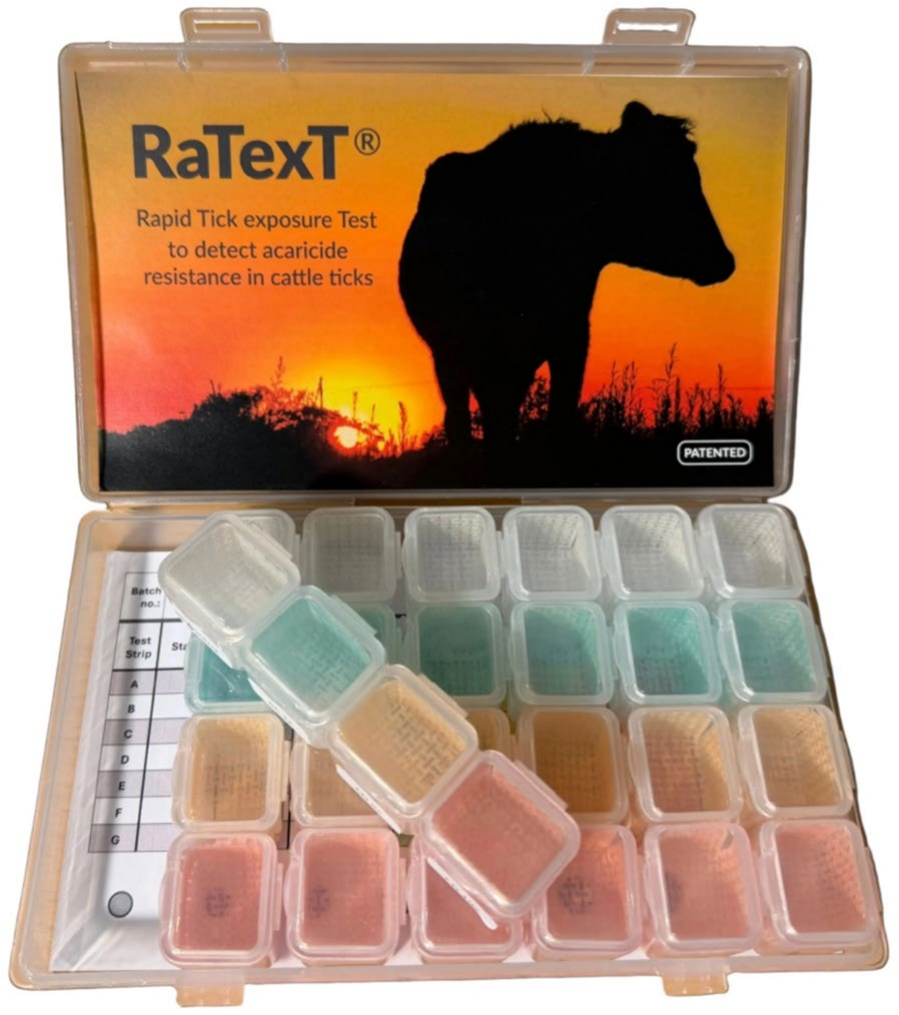
The ultimate design of the rapid tick exposure test (RaTexT) box, comprising seven strips with four interconnected compartments. A single strip has been shown in this study to be sufficient for detecting resistance in livestock ticks

An overall agreement of 91.3% between individual strips and their corresponding RaTexT® boxes containing six strips indicated a high level of consistency between replicate results. Mixing ticks removed from a cattle herd at a particular farm into a shallow container before loading them into RaTexT® boxes may have contributed to a more randomised sampling strategy and partly explains the high level of consistency between replicate strips. The predefined threshold of ≥ 90% accuracy in the RaTexT®, consistently used in our previous work with field-collected cattle ticks in Brazil and East Africa [33, 37], was met with only one strip, supporting the robustness of the test even with minimal replication. However, small but consistent gains in accuracy were observed when three strips were used, which is an attractive option in situations where the number of ticks collected is sufficient. Bootstrap simulations (1000 replicates) showed that three strips yielded 94.9% accuracy (SD 2.07%). Increasing the number of strips could help mitigate potential misclassification arising from technical variability or borderline results. Although further gains could be achieved with all six strips to reach an overall agreement of 96.8% (SD 0.00%) (Fig. 1), using a whole box at a single farm becomes prohibitively counterproductive. Conversely, single test strips will significantly enhance the uptake of RaTexT® in the field, reducing labour and resulting in substantial cost savings. Figure 5 illustrates the final design of the RaTexT box, which comprises seven strips with four interconnected compartments; a single strip has been shown in this study to be sufficient for detecting resistance in livestock ticks.

The next question centered on the optimal number of ticks per test compartment. To determine this, Monte Carlo simulations assessing the effect of the number of ticks per compartment revealed that diagnostic accuracy steadily improved from 81.2% with five ticks to 86.1% with eight ticks, suggesting that seven to eight ticks per compartment offer a practical balance between test efficiency and diagnostic stability (Fig. 2).

The internal CV analysis offered insight into the reproducibility within individual boxes and highlights differences in response between acaricides. Cypermethrin/chlorpyriphos/PBO displayed lower CVs at 5× CD (0.305) and 10× CD (0.194), indicating more consistent mortality responses among strips at higher doses. Conversely, deltamethrin showed consistently higher CVs across all tested doses (1× RD = 1.285; 5× CD = 1.109; 10× CD = 1.268), suggesting greater variability in tick responses, possibly due to differences in individual susceptibility. Furthermore, the high variability observed in the control groups for both compounds emphasised the importance of procedural consistency (such as uniform tick size and vigour/fitness), as minor deviations during test setup may disproportionately affect control mortality.

Taken together, these findings demonstrate that RaTexT® provides high diagnostic accuracy and consistent agreement between single strips and full-box classifications. They also show that internal repeatability, as measured by CV, varies among acaricides. For deltamethrin, the higher CV values can be attributed to a combination of biological and statistical factors. Widespread knockdown resistant genotypes likely push most populations towards a highly resistant phenotype, leading to low overall mortality [43]. In such conditions, occasional deaths, possibly linked to susceptible heterozygous individuals, have a disproportionate impact, increasing the relative variation. Conversely, organophosphates display lower CV values, consistent with their slower toxicodynamic profile and the lower resistance levels observed in some tested populations. Gradual mortality over time tends to homogenise responses across strips, thereby reducing internal variability. These interpretations remain hypothetical, as no genotyping or mechanistic assays were conducted to directly confirm the role of resistance alleles or metabolic factors in the observed patterns.

Furthermore, the reproducibility between independent observers was high, with an overall agreement of 83.3%. Cohen’s kappa coefficient (*κ* = 0.664) and Gwet’s AC1 (0.707) both indicated substantial agreement according to the Landis and Koch [39] scale. The fact that most discrepancies between observers were limited to adjacent categories (e.g. low vs. moderate resistance) suggests that variability had minimal influence on the final diagnostic outcome.

In comparing the three bioassays (RaTexT®, RIT and AIT), the analyses revealed a clear contrast in assay performance between deltamethrin and cypermethrin/chlorpyrifos/PBO. For deltamethrin, all three assays produced statistically comparable mortality estimates, indicating strong agreement across bioassay methods. This consistency likely reflects the rapid toxicodynamic profile of pyrethroids, where acute knockdown and mortality are readily detected regardless of assay design. The high level of resistance was also confirmed with the LPT, showing elevated resistance ratios estimated for the synthetic pyrethroid in *R. microplus* strains Biotech and UFRRJ (RR 33.8 and 39.5, respectively). In contrast, for the cypermethrin/chlorpyrifos/PBO formulation, the assays diverged: the RaTexT® consistently recorded lower mortalities than the AIT, whereas the RIT recorded significantly higher mortalities. These discrepancies are consistent with the slower toxicological action of organophosphates, which are more effectively captured in the delayed post-treatment readout of AIT, and with larvae used by the RIT. This discrepancy should not necessarily be interpreted as low specificity of the RaTexT®, but rather as a consequence of the different exposure modalities used in the assays. In the AIT, ticks are immersed in freshly prepared aqueous dilutions of commercial formulations for 5 min, ensuring full and uniform contact with the active ingredient under optimised laboratory conditions. In the RaTexT®, however, ticks are exposed to nylon matrix impregnated with pour-on formulations, which more closely mimic field application but may alter absorption and bioavailability, particularly for compounds formulated in oily vehicles. Moreover, larval-based assays such as RIT are inherently more sensitive, as larvae are physiologically more susceptible to acaricide exposure than adults. These methodological differences help explain the divergent outcomes observed for the combination of cypermethrin/chlorpyrifos/PBO. Rather than reflecting intrinsic flaws of any single method or identifying a single assay as the most accurate representation of field reality, the divergence illustrates the complementary perspectives each assay offers: RaTexT® captures a more field-relevant exposure scenario, AIT reflects maximal potential efficacy under optimised laboratory conditions and RIT provides a sensitive population-level screen. Integrating data across assays, therefore, strengthens resistance diagnosis and improves interpretation across acaricide classes. These descriptive outcomes align with the comparative GLM analyses focused on female mortality, reinforcing high pyrethroid resistance and a moderate (UFRRJ) versus low (Biotech) resistance to the combination of cypermethrin with chlorpyriphos.

The rapid test has been introduced in regions with high levels of acaricide resistance, such as Brazil and South Africa, through practical workshops. A practical spreadsheet for determining resistance intensity is provided as supplementary material.

 In conclusion, the present study demonstrates that RaTexT® is a robust, reproducible and operationally practical tool for pen-side detection of acaricide resistance in *R. microplus*. High accuracy at the single-strip level, coupled with substantial inter-observer agreement and predictable gains in precision with limited replication, underscores the reliability of the test under routine field conditions. While internal repeatability varied between acaricides, reflecting underlying biological and toxicodynamic differences, the overall diagnostic performance remained consistent and aligned with outcomes from established laboratory assays. Importantly, RaTexT® provided resistance classifications that were coherent with AIT, RIT and LPT, particularly for pyrethroids, while also revealing assay-specific differences that highlight the complementary nature of these methods. Collectively, these results reinforce the value of the RaTexT® as a rapid, cost-effective approach that bridges the gap between laboratory-based diagnostics and real-world decision-making. Future efforts to automate result interpretation and expand validation across additional acaricide classes and geographic regions will further enhance its applicability as a scalable tool for resistance surveillance and sustainable tick control programs.

## Data Availability

The paper provides the complete dataset used in this study. RaTexT ® is a registered trademark of TBD International, which also holds the international patent.

## Abbreviations

AIT: Adult immersion test
LPT: Larval packet test
PBO: Piperonyl butoxide
RaTexT®: Rapid tick exposure test
RIT: Resistance intensity test
RR: Resistance ratio
